# Exploring sensory sensitivity, cortical excitability, and habituation in episodic migraine, as a function of age and disease severity, using the pattern-reversal task

**DOI:** 10.1186/s10194-023-01618-w

**Published:** 2023-08-07

**Authors:** Angela Marti-Marca, Adrià Vilà-Balló, Xim Cerda-Company, Nara Ikumi, Marta Torres-Ferrus, Edoardo Caronna, Victor J. Gallardo, Alicia Alpuente, Mireia Torralba Cuello, Salvador Soto-Faraco, Patricia Pozo-Rosich

**Affiliations:** 1 Headache and Neurological Pain Research Group, Vall d’Hebron Institute of Research (VHIR), Department of Medicine, Universitat Autonoma Barcelona, Barcelona, Spain; 2 Headache Unit, Department of Neurology, Hospital Universitari Vall d’Hebron, Barcelona, Spain; 3 Multisensory Research Group, Center for Brain and Cognition, Pompeu Fabra University, 08005 Barcelona, Spain; 4 Catalan Institution for Research and Advanced Studies (ICREA), 08010 Barcelona, Spain

**Keywords:** Migraine, EEG, Visual processing, Visual sensitivity, Cortical excitability, Habituation, Pattern-reversal visual evoked potentials, Event-related potentials

## Abstract

**Background:**

Migraine is a cyclic, neurosensory disorder characterized by recurrent headaches and altered sensory processing. The latter is manifested in hypersensitivity to visual stimuli, measured with questionnaires and sensory thresholds, as well as in abnormal cortical excitability and a lack of habituation, assessed with visual evoked potentials elicited by pattern-reversal stimulation. Here, the goal was to determine whether factors such as age and/or disease severity may exert a modulatory influence on sensory sensitivity, cortical excitability, and habituation.

**Methods:**

Two similar experiments were carried out, the first comparing 24 young, episodic migraine patients and 28 healthy age- and gender-matched controls and the second 36 middle-aged, episodic migraine patients and 30 healthy age- and gender-matched controls. A neurologist confirmed the diagnoses. Migraine phases were obtained using eDiaries. Sensory sensitivity was assessed with the Sensory Perception Quotient and group comparisons were carried out. We obtained pattern-reversal visual evoked potentials and calculated the N1-P1 Peak-to-Peak amplitude. Two linear mixed-effects models were fitted to these data. The first model had Block (first block, last block) and Group (patients, controls) as fixed factors, whereas the second model had Trial (all trials) and Group as fixed factors. Participant was included as a random factor in both. N1-P1 first block amplitude was used to assess cortical excitability and habituation was defined as a decrease of N1-P1 amplitude across Blocks/Trials. Both experiments were performed interictally.

**Results:**

The final samples consisted of 18 patients with episodic migraine and 27 headache-free controls (first experiment) and 19 patients and 29 controls (second experiment). In both experiments, patients reported increased visual hypersensitivity on the Sensory Perception Quotient as compared to controls. Regarding N1-P1 peak-to-peak data, there was no main effect of Group, indicating no differences in cortical excitability between groups. Finally, significant main effects of both Block and Trial were found indicating habituation in both groups, regardless of age and headache frequency.

**Conclusions:**

The results of this study yielded evidence for significant hypersensitivity in patients but no significant differences in either habituation or cortical excitability, as compared to headache-free controls. Although the alterations in patients may be less pronounced than originally anticipated they demonstrate the need for the definition and standardization of optimal methodological parameters.

## Background

Migraine is often characterized as a cyclic, neurosensory disorder due to reports of altered sensory processing in both ictal (migraine attack) and interictal (attack-free) phases [1, 2]. Although sensory alterations have been observed in different modalities, the visual one remains the most highly researched [3, 4]. Furthermore, aside from the presence of ictal photophobia as a criterion for the diagnosis of migraine [5] visual alterations have also been reported interictally.

Previous studies exploring whether sensory processing is altered in migraine have focused on different processes. The first of these, sensory sensitivity, or hypersensitivity (i.e., a heightened perception or discomfort) to a variety of stimuli and stimulus characteristics, has traditionally been measured using self-report questionnaires [6–8] and sensory thresholds [9–12]. In the interictal phase, patients with migraine have been found to report an increased number of visual stressors in their environment, including a heightened sensitivity to glare, flicker, and contrasting patterns [6, 13] as well as decreased visual thresholds [10, 14, 15]. One of the questionnaires used to assess sensory sensitivity is the Sensory Perception Quotient (SPQ), which evaluates a variety of visual parameters and has been used in both healthy and clinical populations [16].

Another process, which has been assessed to understand sensory alterations in migraine is cortical excitability, with reports of abnormality (both hyper- and hypoexcitability), observed through either increased or decreased amplitudes of pattern-reversal visual evoked potentials (PR-VEPs) [17] (for a review see [18]). In fact, the combination of electroencephalography (EEG) and the Pattern-Reversal (PR) task (e.g., [18–21]), is frequently used to study visual processing, in both clinical and research applications [22]. This task consists of a black and white checkerboard with a given spatial frequency, which reverses its pattern at a predefined temporal frequency. The abrupt onset/offset stimulation constitutes a powerful tool to elicit visual evoked potentials including N1 (also referred to as N70, N75, N80) and P1 (P100). Both components have their maximum amplitude at posterior sites. N1 is a negative component peaking around 75 ms after stimulus onset/offset, sensitive to stimulus characteristics such as contrast [23], spatial frequency [24], stimulus salience, and the degree of attention [25]. P1, is a positive component, peaking around 100 ms, related to luminance [26] and contrast [23], and also modulated by stimulus unpleasantness [27]. Many studies using the PR task in migraine use a peak-to-peak difference amplitude measure (N1-P1) as an index of visual processing, given its correlation to psychophysical measures and to avoid the distortion of the amplitude of later components, such as P1, due to the earlier components, such as N1 [28]. In particular, the N1-P1 amplitude at the beginning of the experiment has frequently been used to assess cortical excitability [29]. Some studies found a decreased amplitude [30] whereas others reported an increased one [31, 32], in patients with migraine as compared to headache-free controls (despite [17, 33–35], for a review of the literature, see Table 1).

**Table 1:** A summary list of studies, in patients with migraine, related to cortical excitability and specifically event-related potential (ERP) component latencies, amplitudes, and peak-to-peak amplitude differences in response to Pattern-Reversal stimulation

**Reference**	**No of subjects and diagnosis**	**Mean age** ± **SD (range)**	**Session timing**	**Spatial freq**	**Temporal freq**	**No of trials **	**ERPs**	**Direction of results and principal findings (group differences)**	
Kennard et al. [36]	28 MA 30 HC	39 ?	NA	NA	2 Hz	256	N1, P1, N2		**L, A (P1) in MA compared to HC**
Benna et al. [37]	10 MwoA 10 with TIA	36 (25–46) 48 (44–52)	At least 8 days after attack	NA	NA	NA	N80, P1	N.S. (latency or amplitude)	
Polich et al. [38]	20 MA 20 HC	33 ± 7 (23–44) ?	Headache-free at test	16 × 16	3.9 Hz	200 × 2	N75, P1, N145 N75-P1	N.S. (latency or amplitude)	
Mariani et al. [39]	22 MwoA 20 HC	39 ± 11 (17–60) 40 ± 12 (21–60)	At least 48 h after attack	38'	1 Hz	128 × 2	P1 N1-P1	N.S. (latency or amplitude)	
Raudino [40]	34 MwoA 6 MA 20 HC	M women: 37 (17–78) M men: 30 (14–43) HC women: 38 (17–54) HC men: 37 (19–55)	Headache-free at test	NA	1.5 Hz	NA	P1	N.S. (latency or amplitude)	
Diener et al. [41]	54 MwoA 4 MA 87 HC	42 35	NA	60' × 60'	1.56 Hz 8.33 Hz	64	P1 and (N1-Pl) + (N2-P1)/2		**L, A (P1) in M compared to HC**
Lai et al. [42]	25 MA 13 MwoA	29 [median] (17–38)	Not specified	27.6'	NA	128	N1, P1 N1-P1	N.S. (latency or amplitude)	
Drake et al. [43]	50 MwoA 37 HC	? (16–67)	NA	56'	1.88 Hz	200 × 2	N1, P1, N2	N.S. (latency or amplitude)	
Mariani et al. [44]	20 MA 20 HC	34 ± 12 (19–55) 37 ± 10 (21–51)	At least 48 h after attack	38'	1 Hz	128 × 2	P1 N75-P1		**L (P1) in MA compared to HC**
Tsounis et al. [45]	22 MwoA 22 MA 37 HC	37 (15–56) 32 (18–58)	At least 2 weeks after attack	49'	1 Hz	128 × 2	P1	N.S. (latency or amplitude)	
Tagliati et al. [46]	7 MwoA 8 visual prodromes 15 HC	32 ± 9 (17–56) ? (18–50)	At least 1 week after attack	NA	NA	240	N70, P1 N70-P1	N.S. (latency or amplitude)	
Schoenen et al. [19]	27 MwoA 9 MA 16 HC	32 33	At least 1 week after attack	8'	3.1 Hz	50 × 5	N1, P1, N2 N1-P1 P1-N2	N.S. (latency or amplitude)	
Shibata et al. [47]	14 MwoA 19 MA 43 HC	40 (20–62) 42 (20–70) 41 (18–71)	At least 2–20 days after attack	30'	1 Hz	100 × 2	N75, P1, N145 N75-P1 P1-N145		**A (N75-P1) in MA compared to HC**
Sener et al. [48]	23 MwoA 16 MA 17 HC	33 ± 7 36 ± 9	At least 1 week after attack	NA	2 Hz	200 × 2	P1 N70-P1	N.S. (latency or amplitude)	
Shibata et al. [49]	14 MwoA 15 MA 23 HC	40 (22–65) 46 (22–65) 43 (20–65)	At least 5 days after attack	30'	2 Hz	100 × 2	P1 N75-P1		**A (N75-P1) in MA compared to HC**
Afra et al. [20]	25 MwoA 15 MA 25 HC	36 30	At least 5 days after attack	8'	3.1 Hz	100 × 15	N1, P1, N2 N1-P1 P1-N2	N.S. (latency or first block amplitude)	
Shibata et al. [50]	20 MA 19 ME (aura, no headache) 34 HC	41 (22–68) 48 (22–70) 48 (20–72)	At least 1–30 days after attack	30'	2 Hz	100 × 2	P1 N75-P1		**A in MA and ME compared to HC**
Oelkers et al. [31]	13 MwoA 13 MA 28 HC	29 ± 6 27 ± 4	± 3 days before and after attack	0.5 c.p.d 1 c.p.d 2 c.p.d 4 c.p.d	1 Hz	50 × 5 per cond	N1, P1, N2 N1-P1 P1-N2		**L (N2, 2, 4 c.p.d.)** **A (P1-N1, 0.5 c.p.d.) in MA and MwoA compared to HC**
Wang et al. [33]	22 MwoA 13 ETH 20 CTH 26 HC	35 ± 10 27 ± 11 28 ± 8 32 ± 12	At least 1 week after attack	8'	3 Hz	50 × 5	N1, P1, N2 N1-P1 P1-N2	N.S. (latency or first block amplitude)	
Afra et al. [34]	12 MA 10 HC	34 ± 16 28 ± 6	± 3 days before and after attack	68'	3.1 Hz	50 × 5	N1, P1 N1-P1	N.S. (latency or amplitude)	
Afra et al. [17]	37 MwoA 22 MA 23 HC	36 ± 11 27 ± 7	± 3 days before and after attack	68'	3.1 Hz	50 × 5	N1, P1 N1-P1	N.S. (latency or first block amplitude)	
Yücesan et al. [51]	49 MwoA (Group 1, migraine duration of 2 years or less; Group 2, migraine duration of at least 10 years) 17 HC	29 ± 8 (18–48) 37 ± 7 (23–52) 36 ± 9 (18–48)	At least 1 week after attack	NA	2 Hz	250 × 2	P1 N70-P1	N.S. (latency or amplitude)	
Judit et al. [52]	69 MwoA 4 MA 4MwoA + MA 23 HC	34 35	± 3 days before and after attack (37 MwoA) 1 day before (8 M) during (15 M) 1–2 days after (32 M)	68'	3.1 Hz	50 × 5	N1, P1 N1-P1	N.S. (latency or amplitude)	
Khalil et al. [53]	47 MA 37 MwoA 8 MwoA + MA 62 HC	40 ± 13 (16–59) 37 ± 13 (17–58)	Headache-free at test	37.8'	2 Hz	240	P1		**L, A (P1) in M compared to HC**
Sand & Vingen [35]	6 MA 15 MwoA 22 HC	39 ± 9 40 ± 9	Preattack group: headache 24 h after (8 M) Interictal: no headache after (13 M)	8' 33'	2 Hz	100 × 2	N70, P1, N145 N70-P1 P1-N145	N.S. (amplitude)	
Logi et al. [54]	40 MwoA 19 MA 30 HC	36 ± 14 38 ± 10	At least 10 days after attack	14.3'	1 Hz	100 × 2 or × 3	N70, P1 P60-N70 N70-P1	N.S. (latency or amplitude)	
Yilmaz et al. [55]	16 MwoA 29 MA 22 HC	32 (11–64) 34 (15–60)	During (26 M) Between (19 M) attacks	NA	2 Hz	200	N1, P1, N2 N1-P1		**L (N2) in MA compared to HC**
Kochar et al. [56]	25 M ? HC	NA	During and 7 days after attack	NA	NA	NA	P1		**L (P1) in M (ictal)**
Ozkul and Bozlar [57]	44 MwoA 35 MA 40 HC	36 ± 10 34 ± 9 33 ± 8	± 3 days before and after attack	68'	3.1 Hz	50 × 5	N1, P1 N1-P1	N.S. (latency or first block amplitude)	
Coutin-Churchman & de Freytez [58]	24 MA 50 HC	? (18–53) ? (18–47)	Interictal (not specified)	30'	2 Hz	100 × 2	P1 N1-P1 P1-N2		**A in MA compared to HC** **N.S. (latency)**
Ashjazadeh & Varavipour [59]	27 MA 26 MwoA 55 HC	(15–57) (15–48)	At least 1 week after attack	NA	2 Hz	200 × 2	N75, P1, N145 P1-N145		**L (P1) in MA compared to HC**
Spreafico et al. [60]	19 MA 34 MwoA 20 HC	38 35	At least 5 days after the last attack	240.5'	3 Hz	100 × 15	P1		**L (P1) in M compared to HC**
Coppola et al. [61]	27 MA 20 MA + 30 HC	32 ± 9 33 ± 10 33 ± 13	± 3 days before and after attack	15'	1.55 Hz	100 × 6	N1, P1, N2 N1-P1 P1-N2		**Last block A (N1-P1) in MA compared to HC** N.S. (latency)
Coppola et al. [62]	15 MwoA 15 MA 15 HC	31 ± 10 30 ± 10 28 ± 8	± 3 days before and after attack	15'	3.1 Hz	100 × 6	N1, P1 N1-P1	N.S. (latency or amplitude)	
Di Clemente et al. [63]	15 MwoA 15 HC	28 ± 11 24 ± 3	± 2 days before and after attack	68'	3.1 Hz	100 × 6	N1, P1 N1-P1	N.S. (latency or first block amplitude)	
Shibata et al. [24]	14 MwoA 11 MA 25 HC	41 ± 11 44 ± 15 40 ± 10	± 72 h before and after attack	0.5 c.p.d 1.0 c.p.d 4.0 c.p.d	1 Hz	100 × 2	N75, P1, N135 P50-N75 N75-P1 P1-N135		**L (N135, 4.0 c.p.d) in M compared to HC** **A (P50-N75, 1.0 c.p.d; P1-N135, 4.0 c.p.d.) in M compared to HC**
Sand et al. [21]	33 MwoA 8 MA 31 HC	37 ± 13 37 ± 16 40 ± 11	Preattack (13 M) Attack (13 M) Postattack (10 M) Interictal (± 72 h before and after attack: (all M)	31' 62'	0.95 Hz	50 × 4	N1-P1 P1-N2		**A (N1-P1) in MA compared to MwoA, HC** **A (P1-N2) in pre-attack compared to interictal; and MA compared to MwoA, HC**
Sand et al. [32]	33 MwoA 8 MA 31 HC	37 ± 13 37 ± 16 40 ± 11	Preattack (13 M) Interictal (± 72 h before and after attack)	31' 62'	0.95 Hz	50 × 4	N1-P1 P1-N2		**A (P1-N2, medium and large checks) in MA compared to MwOA** **A (N1-P1, large checks) in MA compared to MwoA** N.S. A (M vs. HC)
Marinis et al. [64]	40 MA 40 MwoA 40 HC	32 ± 8 32 ± 9 32 ± 8	At least 72 h after the last attack	38'	1 Hz	100 × 2	P1	N.S. (latency)	
Shibata et al. [65]	10 MA 10 MwoA 20 HC	39 (20–57) 41 (20–58) 39 (20–60)	At least 72 h after and 48 h before an attack	0.5 c.p.d 2.0 c.p.d	5 Hz 10 Hz	6 to 15 s each	Ampl. and phase of 2^nd^ and 4^th^ harmonic (2F and 4F)		**2F: A (0.5 c.p.d.) in M compared to HC**
									**4F: A (2.0 c.p.d., 10 Hz, high contrast) in MA compared to MwoA, HC**
Boylu et al. [66]	41 M ? HC	NA	NA	NA	NA	NA	N75, P1, N145 N75-P1		**L (N75, P1) in M compared to HC** **A, L (N145) in M compared to HC**
Khalil et al. [67]	47 MA 62 HC	16–59	At least 3 days after an attack	38'	2 Hz	240	P1	N.S. (amplitude)	
Nguyen et al. [68]	26 MwoA 19 MA 30 HC	28 ± 6 (20–41) 33 ± 6 (19–43) 26 ± 7 (19–46)	At least 7 days after an attack 4 M had headache 72 h after	48' 15' 960'	1 Hz 8.3 Hz	200 (100 × 2)	N75, P1, N135		**A (P1, 1 Hz) for MA compared to MwoA and HC**
Shibata et al. [69]	12 MwoA 12 MA 12 HC	41 (20–59) 43 (20–60) 42 (20–60)	± 72 h before and after attack	0.5 c.p.d 1.0 c.p.d 2.0 c.p.d 4.0 c.p.d	7.5 Hz	20 × 4	Steady-state VEPs		**A (2.0 c.p.d.) in MA compared to MwoA and HC**
Coppola et al. [70]	21 MwoA 22 MA 22 Mict 21 HC	27 ± 7 31 ± 10 34 ± 12 28 ± 8	Interictal: ± 3 days before and after attack Ictal: ± 12 h around attack	15'	1.55 Hz	100 × 6	N1-P1	N.S. (first block or last block amplitude)	
Omland et al. [71]	12 MA 15 MwoA 34 HC	28 ± 8 31 ± 10	± 48 h before and after attack	8' 65'	1.5 Hz	100 × 6	N70, P1, N145 N70-P1 P1-N145	N.S. (latency or amplitude)	
Di Lorenzo et al. [72]	14 MwoA 4 MA 18 HC	39 (19–54) 39	± 3 days before and after attack	15'	1.55 Hz	100 × 6	N1, P1, N2 N1-P1 P1-N2	N.S. (latency or amplitude)	
Vigano et al. [73]	13 MwoA 11 HC	29 ± 5 26 ± 6	± 72 h before and after attack	15 mm side	3.1 Hz	100 × 6	N1-P1 P1-N2	N.S. (first block amplitude)	
Lisicki et al. [30]	30 M 30 HC (15 with a first-degree relative with M, and 15 without)	27 ± 7 28 ± 9 25 ± 3	± 72 h before and after attack	14'	3.1 Hz	100 × 6	N1-P1		**A (first block) in M and HC with first-degree relatives with migraine compared to HC without** **Negative correlation with habituation slope**
Omland et al. [74]	25 Mint (14 MwoA 11MA) 7 Mpreict (3 MwoA, 4 MA) 32 HC	27 ± 8 27 ± 9 30 ± 10	Pre-ictal: < 48 h before attack Interictal: ± 48 h before and after attack	8' 65'	1.5 Hz	100 × 6	N70-P1 P1-N145	N.S. (amplitude)	
Ambrosini et al. [75]	13 MwoA 15 HC	33 ± 10 (18–55) 30 ± 8 (21–44)	± 3 days before and after attack	68'	3.1 Hz	100 × 6	N1-P1	N.S. (amplitude)	
Coppola et al. [61]	27 MA 20 MA + 30 HC	32 ± 9 33 ± 10 33 ± 13	± 3 days before and after attack	15'	1.55 Hz	100 × 6	N1, P1, N2 N1-P1 P1-N2		**A last block in M compared to HC**
Rauschel et al. [76]	41 M 40 HC	30 ± 10 28 ± 8	± 48 h before and after attack	51'	3 Hz	75 × 6	N75-P1	N.S. (amplitude)	
Omland et al. [77]	24 MwoA 15 both MwoA and MA 2 MA 30 HC	39 ± 10 (19–56) 38 ± 11 (21–59)	± 2 days before and after attack	16'	1.5 Hz	100 × 6	N70, P1, N145 N70-P1 P1-N145	N.S. (latency or first block amplitude)	
Verroiopoulos et al. [78]	15 MA 23 MwoA 20 HC	39 ± 9 48 ± 12 47 ± 11	± 24 h before and after attack	58.8'	1 Hz	200	P1 N80-P1	N.S. (latency or amplitude)	
El-Shazly et al. [79]	60 MA 30 HC	31 ± 3 31 ± 4	± 3 days before and after attack or during aura	60' (48'-72') Small: 16' Large: 64'	1 Hz	100 × 2	N75, P1 N75-P1		**L (P1)** **A (P1) during aura compared to interictal MA, HC**
Kalita et al. [80]	65 M 30 HC	34 ± 12 31 ± 8	Phase not controlled, headache at session noted	12' × 16'	3 Hz	100 × 5	N75, P1	N.S. (first block amplitude)	
Susvirkar et al. [81]	40 M 40 HC	21 ± 0.4 21 ± 0.4	± 3 days before and after attack	NA	1 Hz	300 × 4	N75, P1, N145		**L (N75, N145)** **A (P1) in M as compared to HC**
Coppola et al. [82]	19 MA 22 MwoA 14 HC	30 ± 10 29 ± 8 30 ± 6	± 3 days before and after attack; during attack (10 M)	15'	1 Hz	40 × 10	P1 N75-P1	N.S. (latency or amplitude)	
Kalita et al. [83]	91 M 25 HC	32 ± 11 33 ± 11	NA	12' × 16'	3 Hz	100 × 5	N75, P1		**A (P1, first block) in M compared to HC**

Units of measurement: visual angle in minutes of arc ('); cycles per degree (c.p.d); hertz (Hz)

*Abbreviations*: *L* latency, *A* amplitude, *MwoA* migraine without aura, *MA* migraine with aura, *M* migraine, *TIA* transient ischemic attack, *ETH *episodic tension-type headache, *CTH *chronic tension-type headache, *MAtot *total number of patients with migraine with aura, *MA + *complex neurological aura, *HC *headache-free control. Upward facing arrows indicate an increase (in amplitude) and/or a longer latency, whereas downward facing arrows indicate a reduction (in amplitude) and a shorter latency

Bold writing represents a significant group effect

Finally, habituation has been studied in patients with migraine and headache-free controls, and a deficit of habituation or even potentiation of the N1-P1 peak-to-peak amplitude over time (or experimental blocks; final blocks compared to first blocks) has been found in patients with migraine interictally [19, 70]. Despite these findings being proposed as robust, some controversy remains, particularly given the presence of negative or inconsistent results [21, 31, 32, 35] (see Table 2 for a review of the literature).

**Table 2:** A summary list of studies, in patients with migraine, related to habituation and specifically event-related potential (ERP) component latencies, amplitudes, and peak-to-peak amplitude differences in response to Pattern-Reversal stimulation

**Reference**	**No of subjects and diagnosis**	**Mean age ± SD (range)**	**Timing of session**	**Spatial freq**	**Temp. freq**	**No of trials**	**ERPs**	**Principal findings**
Schoenen et al. [19]	27 MwoA 9 MA 16 HC	32 33	At least 1 week after attack	8'	3.1 Hz	50 × 5	N1-P1 P1-N2	**Habituation deficit and potentiation in M** **Normal habituation in HC**
Afra et al. [20]	25 MwoA 15 MA 25 HC	36 30	At least 5 days after attack	8'	3.1 Hz	100 × 15	N1-P1 P1-N2	**Habituation deficit and potentiation in M** **Normal habituation in HC**
Wang et al. [33]	22 MwoA 13 ETH 20 CTH 26 HC	35 ± 10 27 ± 10 28 ± 8 32 ± 12	At least 1 week after attack	8'	3 Hz	50 × 5	N1-P1 P1-N2	**Habituation deficit and potentiation in M** **Normal habituation in ETH, CTH, and HC**
Oelkers et al. [31]	13 MwoA 13 MA 28 HC	29 ± 6 27 ± 4	± 3 days before and after attack	0.5 c.p.d 1.0 c.p.d 2.0 c.p.d 4.0 c.p.d	1 Hz	50 × 5	N1-P1 P1-N2	N.S. in habituation
Sándor et al. [84]	40 MwoA (20 parents and their children)	44 ± 8 17 ± 6	± 3 days before and after attack	8'	3.1 Hz	50 × 5	N1, P1	**Habituation deficit patterns in related migrainous pairs**
Áfra et al. [34]	12 MA 10 HC	34 ± 16 28 ± 6	± 3 days before and after attack	68'	3.1 Hz	50 × 5	N1-P1	N.S. in habituation
Áfra et al. [17]	37 MwoA 22 MA 23 HC	36 ± 11 27 ± 7	± 3 days before and after attack	68'	3.1 Hz	50 × 5	N1-P1	**Habituation deficit and potentiation in M** **Normal habituation in HC** **Negative correlation between 1** ^**st**^ **block amplitude and habituation**
Judit et al. [52]	69 MwoA 4 MA 4MwoA + MA 23 HC	34 35	± 3 days before and after attack (37 MwoA) 1 day before (8 M) during (15 M) 1–2 days after attack (32 M)	68'	3.1 Hz	50 × 5	N1-P1	**Habituation deficit in M interictally, normalization just before and during attack**
Sand & Vingen [35]	6 MA 15 MwoA 22 HC	39 ± 9 40 ± 9	Preattack group: headache 24 h after session (8 M) Interictal: no headache after (13 M)	8' 33'	2 Hz	100 × 2	N70-P1 P1-N145	N.S. in habituation (8' or 33' checks) No habituation of either M or HC to 33' checks
Bohotin et al. [85]	20 MwoA 10 MA 24 HC	34 ± 10 24 ± 3	± 3 days before and after attack	8'	3.1 Hz	100 × 6	N1-P1 P1-N2	**Habituation deficit and potentiation in M before rTMS** **Normal habituation in HC**
Ozkul & Bozlar [57]	44 MwoA 35 MA 40 HC	36 ± 10 34 ± 9 33 ± 8	± 3 days before and after attack	68'	3.1 Hz	50 × 5	N1-P1	**Habituation deficit and potentiation in M** **Normal habituation in HC** **Negative correlation between 1** ^**st**^ **block amplitude and habituation for HC and MwoA**
Di Clemente et al. [63]	15 MwoA 15 HC	28 ± 11 24 ± 3	± 2 days before and after attack	68'	3.1 Hz	100 × 6	N1-P1	**Habituation deficit and potentiation in M** **Normal habituation in HC**
Coppola et al. [62]	15 MwoA 15 MA 15 HC	31 ± 10 30 ± 10 28 ± 8	± 3 days before and after attack	15'	3.1 Hz	100 × 6	N1-P1	**Habituation deficit and potentiation in M** **Normal habituation in HC**
Fumal et al. [86]	6 MwoA 2 MA 8 HC	23 ± 1 23 ± 2	± 3 days before and after attack	8'	3.1 Hz	100 × 6	N1-P1	**Habituation deficit and potentiation in M** **Normal habituation in HC**
Magis et al. [87]	24 MwoA 28 MA	32 ± 14	72 h before and after attack)	51′ 33''	3.1 Hz	100 × 6	N1-P1	**Habituation deficit in M is more marked in patients with no mutation of MTHFR C677T polymorphism compared to homozygous** **Lower first block amplitude correlated to greater habituation deficit**
Sand et al. [21]	33 MwoA 8 MA 31 HC	37 ± 13 37 ± 16 40 ± 11	Preattack (13 M) Attack (13 M) Postattack (10 M) Interictal (± 72 h before and after attack; all M)	31' 62'	0.95 Hz	50 × 4	N1-P1 P1-N2	N.S. in habituation (62’)
Sand et al. [32]	33 MwoA 8 MA 31 HC	37 ± 13 37 ± 16 40 ± 11	Preattack (13 M) Interictal (± 72 h before and after attack)	31' 62'	0.95 Hz	50 × 4	N1-P1 P1-N2	N.S. in habituation (62’), neither M or HC habituate
Coppola et al. [88]	18 MwoA 18 HC	31 27	± 3 days before and after attack	15'	3.1 Hz	100 × 6	N1-P1 P1-N2	**Habituation deficit and potentiation in M** **Normal habituation in HC**
Coppola et al. [89]	12 MwoA 19 HC	28 ± 6 26 ± 4	± 3 days before and after attack	15'	3.1 Hz	100 × 6	N1-P1	**Habituation deficit and potentiation in M** **Normal habituation in HC**
Coppola et al. [90]	17 MwoA 17 HC	29 ± 12 29 ± 11	± 3 days before and after attack	15'	3.1 Hz	100 × 6	N1-P1	**Habituation deficit and potentiation in M** **Normal habituation in HC**
Hansen et al. [91]	9 FHM 7 HC	38 (20–63) 29 (28–31)	Headache-free at recording	68'	3.1 Hz	100 × 6	N1-P1	**Habituation deficit in FHM** **No habituation in HC**
Shibata et al. [69]	12 MwoA 12 MA 12 HC	41 (20–59) 43 (20–60) 42 (20–60)	± 72 h before and after attack	0.5 c.p.d 1.0 c.p.d 2.0 c.p.d 4.0 c.p.d	7.5 Hz	20 × 4	Steady-state VEPs	N.S. in habituation, HC did not habituate and M no habituation deficit
Coppola et al. [70]	21 MwoA 22 MA 22 Mict 21 HC	27 ± 7 31 ± 10 34 ± 12 28 ± 8	Interictal: ± 3 days before and after attack Ictal: ± 12 h before or after	15'	1.55 Hz	100 × 6	N1-P1	**Habituation deficit and potentiation in MwoA and MA interictallyNormal habituation in HCand Mict**
Omland et al. [71]	15 MwoA 12 MA 34 HC	27 ± 8 31 ± 10	± 48 h before and after attack	8'65'	1.5 Hz	100 × 6	N1-P1P1-N2	**N.S. in habituation (normal in both groups) **
Vigano et al. [73]	13 MwoA11 HC	29 ± 5 26 ± 6	± 72 h before and after attacK	15 mm side	3.1 Hz	100 × 6	N1-P1P1-N2	**N.S. in habituation (N1-P1)Habituation deficit of P1-N2 in MwoA**
Bednar et al. [92]	39 M 36 HC	41 ± 11 (18–62) 37 ± 12 (18–62)	Interictal: ± 72 h before and after attack (19 M) Ictal (10 M)	13'	2 Hz	60 × 5	N75-P1 P1-N145	**Habituation deficit and potentiation in M interictally, ictally, and during treatment compared to HC**
Omland et al. [74]	25 Mint (14 MwoA, 11MA) 7 Mpreict (3 MwoA, 4 MA) 32 HC	27 ± 8 27 ± 9 30 ± 10	Pre-ictal: < 48 h before attack Interictal: ± 48 h before and after attack	8' 65'	1.5 Hz	100 × 6	N70-P1 P1-N145	N.S. in hab between HC and Mint
Ambrosini et al. [75]	13 MwoA 15 HC	33 ± 10 (18–55) 30 ± 8 (21–44)	± 3 days before and after attack	68'	3.1 Hz	100 × 6	N1-P1	**Habituation deficit and potentiation in MwoA** **Normal habituation in HC**
Coppola et al. [61]	27 MA 20 MA + 30 HC	32 ± 9 33 ± 10 33 ± 13	± 3 days before and after attack	15'	1.55 Hz	100 × 6	N1-P1 P1-N2	**Habituation deficit (N1-P1) and potentiation in M** **Normal habituation in HC**
Rauschel et al. [76]	41 M 40 HC	30 ± 10 28 ± 8	± 48 h before and after attack	51'	3 Hz	75 × 6	N75-P1	**Habituation deficit and potentiation in M** **Normal habituation in HC**
Ambrosini et al. [93]	624 EM 439 MwoA 185 MA 360 HC	(25–37)	± 3 days before and after attack	68' 15'	3.1 Hz	50 × 5 100 × 6	N1-P1	**Habituation deficit and potentiation in M** **Normal habituation in HC**
Omland et al. [77]	24 MwoA 15 both MwoA and MA 2 MA 30 HC	39 ± 10 (19–56) 38 ± 11 (21–59)	± 2 days before and after attack	16'	1.5 Hz	100 × 6	N70-P1 P1-N145	N.S. in habituation (normal in both groups)
Verroiopoulos et al. [78]	15 MA 23 MwoA 20 HC	39 ± 8 48 ± 12 47 ± 11	± 24 h before and after attack	58.8'	1 Hz	200	N80-P1	N.S. in habituation (normal in both groups)
Di Lorenzo et al. [72]	14 MwoA 4 MA 18 HC	39 (19–54) 39	± 3 days before and after attack	15'	1.55 Hz	100 × 6	N1-P1 P1-N2	**Habituation deficit and potentiation in M** **Normal habituation in HC**
Lisicki et al. [30]	30 M 30 HC (15 with a first-degree relative with M, and 15 without)	27 ± 7 28 ± 9 25 ± 3	± 72 h before and after attack	14'	3.1 Hz	100 × 6	N1-P1	**Habituation deficit and potentiation in M and HC with relative with migraine** **Normal habituation in HC without first-degree relatives**
Ince et al. [94]	52 M 35 HC	36 ± 9 34 ± 10	± 3 days before and after attack	NA	3.1 Hz	100 × 10	N1-P1 P1-N2	**Habituation deficit in M** **Normal habituation in HC**
Kalita et al. [80]	65 M 30 HC	34 ± 12 31 ± 8	Phase not controlled, presence of headache noted	12 × 16'	3 Hz	100 × 5	N75, P1	**Habituation deficit (N75) M** **Normal habituation in HC**
Lisicki et al. [95]	25 MwoA	26 ± 6	± 72 h before and after attack	14'	3.1 Hz	100 × 6	N1-P1	**Preserved habituation in patients that do not perceive stress as a trigger, habituation deficit in rest of patients**
Susvirkar et al. [81]	40 M 40 HC	21 ± 0.4 21 ± 0.4	± 3 days before and after attack	NA	1 Hz	300 × 4	P1	**Habituation deficit and potentiation in M** **Normal habituation in HC**
Kalita et al. [83]	91 M 25 HC	32 ± 11 33 ± 11	NA	12' × 16'	3 Hz	100 × 5	N75, P1	**Habituation deficit and potentiation in M** **Normal habituation in HC**

Units of measurement: visual angle in minutes of arc ('); angle in seconds of arc (''), cycles per degree (c.p.d); hertz (Hz)

*Abbreviations*: *MwoA* migraine without aura, *MA* migraine with aura, *M* migraine, *EM* episodic migraine, *EMint* episodic migraine interictally, *EMict* episodic migraine ictally, *Mict* migraine during the ictal phase, *Mpreict* migraine in the pre-ictal phase, *CM* chronic migraine, *ETH* episodic tension-type headache, *CTH* chronic tension-type headache, *MAtot* total number of patients with migraine with aura, *MA +* complex neurological aura, *HC* headache-free control

Bold writing represents a significant group effect

The study of sensory sensitivity, cortical excitability, and habituation across the lifespan is relevant for better disease management. At a clinical level it would appear that migraine (not necessarily episodic migraine) incidence tends to peak in the late 30s [96] and then level off (although some studies report increased frequency during perimenopause and menopause) [97]. Furthermore, accompanying symptoms, in particular photophobia and phonophobia would appear to increase with age [98] up until a point after which, in older patients, over the age of 60, there is a reported decrease [99], and diminish with increasing migraine frequency [100]. In terms of sensory sensitivity, sensory thresholds have been shown to increase with older age, indicating decreased sensory sensitivity, usually in the 60s and onwards [101, 102] (for a review see [103]). 

However, this remains inconclusive with regard to migraine symptomatology, given that it appears linked to the degeneration of sensory receptors ([104]; for a review see [105]). Pertaining to cortical excitability and habituation, to the best of our knowledge, no study has directly evaluated, using PR-VEPs their variation as a function of age and migraine frequency. With current data, it is not possible to establish a relationship between sensory sensitivity, cortical excitability, and habituation nor understand exactly how they are associated with measures related to age and migraine frequency.

The present study aimed to assess whether a relationship exists between visual sensitivity, often reported in episodic migraine interictally, and cortical excitability and/or habituation, and whether it might be modulated by age and disease severity. To accomplish this objective, we carried out a research study involving two experiments. Experiment 1 consisted of a sample of young adults with episodic migraine and their headache-free controls whereas Experiment 2 included middle-aged adults with episodic migraine and their headache-free controls. Given the state of the literature, we hypothesized the presence of hypersensitivity in patients with migraine in both experiments [9, 10, 12, 106] as well as, abnormal cortical excitability (hypo- or hyper-) and a deficit of habituation in patients with migraine as compared with headache-free controls [19, 20, 52] (for a review see [18]), although in the case of the latter, we accept that opposing results are entirely plausible. The novelty of our study can be found in its use of two experiments, which allowed us to collect data from patients with episodic migraine and their age- and gender-matched headache-free controls differing in age and headache frequency and a novel trial-by-trial analysis, using linear mixed-effects models (LMMs), which should help capture both individual and temporal variability.

Prior to their participation, all subjects provided written informed consent. See the Ethics approval and consent to participate section within Declarations for more information.

## Experiment 1

### Method

#### Participants

In this experiment, 63 young university students (all females, between 18–30 years old, right-handed, and with normal or corrected-to-normal vision) were included. 35 were diagnosed with episodic migraine (EM) with or without aura by a neurologist, according to the International Classification of Headache Disorders 3rd edition (ICHD-3) [5]. The remaining 28 participants constituted the headache-free control group (HC), which was age- and gender-matched to the EM group. One objective of this experiment was to have a very homogenous and clinically similar sample of participants to reduce biases and effectively compare brain responses. Diagnoses were not formally disclosed to the participants until the end of the study. Exclusion criteria included: known morphological brain abnormalities, neurological or severe psychiatric illness, chronic pain, cardiovascular disease, pregnancy, as well as the use of any pharmaceutical or non-pharmaceutical drugs that could alter the EEG waveform. Patients could not have been previously diagnosed with any other headache disorder and could not have been taking prophylactic medication. Controls could not have had any previous headache diagnosis or first-degree relatives with migraine. Considering previous literature indicating the importance of phase, particularly with regard to the habituation deficit [18], we excluded patients that were outside of the interictal phase (see Results). Specifically, patients who did not report a moderate to severe attack 24 h prior, the day of, and 24 h post-session (72-h headache-free window) were considered interictal (confirmed by a headache virtual daily calendar or eDiary).

#### Procedure and paradigm

Prior to the experimental session, potential participants completed a (A) sociodemographic and anthropometric questionnaire as well as a (B) migraine screening questionnaire based on ICHD-3 [5] criteria. Participants that fit the inclusion/exclusion criteria were subsequently assessed by a neurologist, assigned a diagnosis (EM or HC), and provided with a baseline, virtual, daily headache calendar (eDiary), which was used to obtain an objective measure of headache frequency and confirm interictal phase during the recording. The eDiary also contained questions relative to the presence of headache, its duration, intensity, accompanying symptoms, and acute medication as well as other medication, menses, and participant sleep–wake cycle. All of the participants filled it out for an average of 34 ± 8 days prior to the experimental session, as well as on the day of the recording and at least 24 h after to confirm interictal phase.

The session itself consisted of (i) psychiatric, clinical, and experimental session questionnaires and (ii) an EEG recording. The psychiatric questionnaires included the State-Trait Anxiety Inventory (STAI) [107, 108], ADHD Self-Report Scale (ASRS) [109], and Beck Depression Inventory-II (BDI-II) [110]. To assess sensory sensitivity, we used the SPQ, with lower scores denoting increased sensitivity [16], which evaluates sensory sensitivities across all five modalities and has been validated for use in both healthy adults and clinical populations. On the other hand, clinical questionnaires included the: Headache Impact Test-6 (HIT-6) [111], Migraine Disability Assessment Test (MIDAS) [112], and Migraine-Specific Quality of Life Questionnaire (MSQ) [113]. Participants also completed an experimental session questionnaire which inquired about headache presence and its characteristics, acute medication use, other medication use, sleep quality, fatigue, and menstruation, at the time of the experiment. The questionnaires and eDiary were hosted by Research Electronic Data Capture (REDCap) tools [114, 115], at the Vall d’Hebron Institute of Research.

**Fig. 1:**
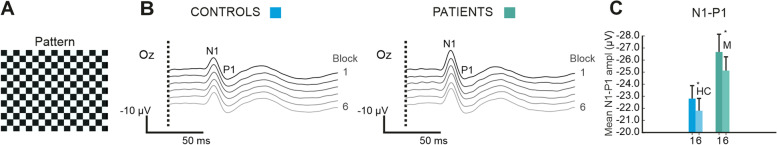
Visual illustrations of the checkerboard pattern and resulting visual evoked potentials (VEPs) and habituation to pattern-reversal (PR) stimulation in Experiment 1. **A** Checkerboard pattern used in the PR task. **B** VEPs at the Oz electrode, with time (in ms) on the x-axis and N1-P1 peak-to-peak amplitude difference voltage on the y-axis, observed for each block (1 to 6) of the Pattern-Reversal task, and both groups (EM right, HC left). **C** Bar graph with Block number on the x-axis and mean N1-P1 peak-to-peak amplitude difference voltage on the y-axis, depicting habituation of the N1-P1 between Blocks 1 and 6 (green EM, blue HC). Please note the decrement in amplitude between the 1st and 6th block

The EEG recording consisted of a 5 min resting state recording followed by the PR task and was performed in a chamber with dimmed lights as well as acoustic and electromagnetic attenuation. Participants sat at a distance of 0.75 m from the computer monitor. The stimuli used for the PR task consisted of a checkerboard pattern of black and white squares (93% contrast; see Fig. 1A). The reversal frequency was set at 1.55 Hz and was based on Coppola et al. whereas the check size or spatial frequency was 6 min of arc (6’), adapted from the recommended 8’ at 1 m [61]. The stimulated visual field was 30.7 cm × 22.5 cm, under binocular presentation. A red fixation point at the center of the screen was present throughout the task to reduce ocular artifacts. Experimental stimuli were programmed and presented, using custom-made scripts, with MATLAB R2017a (The Mathworks Inc., 2017) and Psychophysics Toolbox Version 3.0.13 [116, 117], running on Windows XP. All stimuli were displayed on a Sony Multiscan G520 Trinitron Color Monitor (CRT screen, resolution: 1024 × 768, 120 Hz refresh rate, background luminance: 21 cd/m^2^). Accurate timing of stimuli was confirmed using the Black Box Toolkit (Accuracy of < 0.005 s (seconds); Black Box Toolkit, Ltd., Sheffield, UK). Participants were instructed to remain still and maintain their eyes on the fixation point. The task consisted of 600 trials (3.23 min total duration), segmented into six blocks of 100 trials post-recording.

#### EEG recordings

Continuous EEG recordings (digitized, 500 Hz sampling rate, no online filters) were acquired using a BrainAmp Standard (001 10/2008) amplifier connected to an actiCHamp Control Box (BrainVision Analyzer, Version 2.2.2, Brain Products GmbH, Gilching, Germany). 64 active electrodes (10–10 system) at standard positions were used and an online reference electrode was placed on the tip of the nose whereas a ground electrode was positioned at AFz in the cap. External electrodes consisted of left and right mastoids as well as vertical and horizontal electrooculograms. Impedances were maintained below 15 kΩ.

#### EEG pre-processing

EEG data analyses were performed using EEGLAB 13.5.4b [118] and ERPLAB 7.0.0 [119], as well as MATLAB R2017a (The Mathworks Inc., 2017) custom-made scripts. EEG pre-processing was carried out offline according to standard procedure, which included the use of a 50 Hz notch filter (stop-band Parks-McClellan notch, 180 order). Next, interpolation of noisy channels was done. After, data was band-pass filtered, in two steps, using a Hamming windowed sinc finite impulse response (FIR) filter (zero-phase). First, a high-pass filter of 0.1 Hz (16,501 order, -6 dB cutoff) was applied followed by a low-pass filter of 60 Hz (111 order, -6 dB cutoff). Subsequently, data was segmented to epochs of 0–300 ms time-locked to the reversals and normalized by the mean segment activity, which was re-referenced to the mean-activity of both mastoids. Finally, visual inspection and manual rejection were performed to remove epochs with noise.

#### Analyses

Statistical analyses were carried out using R (R Core Team, 2021, version 4.1.1) and RStudio software (RStudio Team, 2021, version 1.4.1717). The following packages were used: base, car, dgof, dplyr, emmeans, ggpubr, ggResidpanel, graphics, lattice, lme4, nlme, multiplyr, nortest, pgirmess, psych, rstatix, and stats.

##### Psychiatric, clinical, and SPQ questionnaires

Data from the psychiatric, clinical, and SPQ questionnaires were reported using percentages (categorical), means and standard deviations (continuous, normally distributed), or medians and interquartile ranges (continuous, not normally distributed). Group effects were evaluated with Fisher’s exact test, two-sided, unpaired t-tests of equal variance, or two-sided, nonparametric Mann–Whitney U test, respectively.

##### Electrophysiological data

*PR-VEP.* The amplitudes and latencies of N1 and P1 as well as the Peak-to-Peak amplitude difference (N1-P1) were obtained for each trial (1–600), block (1–6), and participant at the Oz electrode (active electrode in [18–20], among others). Only clean trials, free of artifacts, were used. Each block contained a maximum of 100 clean trials, with a mean of 89.46 ± 9.093 (range: 30–100) trials per participant per block. Participants had a grand mean of 536.78 ± 36.962 trials, post artifact rejection, out of a total of 600 (range: 435–595). Components were identified based on visual inspection and peak latencies (reversal-locked) with N1 being the most negative peak between 65–95 ms (peak: 80 ms; window: ± 15 ms) and P1 being the most positive peak between 86–126 ms (peak:106 ms; window: ± 20 ms). The amplitudes of P1 and N1 used to calculate the N1-P1 peak-to-peak were extracted using an automatic system and subsequent visual inspection [61].

*Classic block analyses.* The first series of analyses were comprised of classic block analyses on the N1-P1 peak-to-peak amplitude [120]. A type III two-way mixed analysis of variance (ANOVA) was used with Block (1 and 6) as the between-subject factor and Group (EM and HC) as the within-subject factor. In the event that post hoc tests were necessary, pairwise comparisons were executed, and Bonferroni-adjusted *p* values were obtained (p_adj_).

*Block linear mixed-effects model.* LMMs were fitted to N1-P1 data, using the *nlme* package in R [121], to evaluate cortical excitability and habituation. The fixed effects variables were Block (1 and 6) and Group (EM and HC), and the random effects variable was Participant. We also tested an autocorrelation structure of order 1, in the form of Participant nested within Trial. To ensure that our model was the best alternative, we ran model comparisons using the Akaike Information Criterion (AIC) and a Chi-square test on the model log-likelihoods (Chisq) [122]. Using the final model, we obtained a type III ANOVA table calculating Kenward-Roger "F" tests with Satterthwaite degrees of freedom, where the within-subject factor was Block and the between-subject factor was Group (*F* and *p*-values were reported). The confidence level was set to 0.95. Visual inspection of residual plots did not reveal deviations from homoscedasticity or normality in any measure. In the presence of a significant Block x Group interaction, estimated marginal means were calculated to do post hoc, pairwise comparisons, and *z* ratios and *p* values were reported. The False Discovery Rate (FDR) was applied to adjust for multiple comparisons.

Cortical excitability was assessed by examining peak-to-peak first block amplitude, with differences in this measure suggesting either hypo- (significantly lower amplitude) or hyper-excitability (significantly greater amplitude) in patients with migraine as compared to headache-free controls [29]. Habituation on the other hand referred to a peak-to-peak amplitude decrement between the first and last block [19, 70] and was evaluated both within- and between-groups. Only data from Blocks 1 and 6 were used given that habituation was defined as the difference in the N1-P1 peak-to-peak amplitude between the first and last block (for a review see Table 5 in [71]). Please note that to perform these comparisons the presence of a Block x Group interaction was necessary.

*Trial linear mixed-effects model.* Using the N1-P1 peak-to-peak data, we also went one step further and fitted LMMs using the *nlme* package in R [121] taking into account trial-by-trial fluctuation thus increasing the precision of our measures. The first model (the Block LMM) was selected to be more similar to past literature (comparing first block and last block measures; see Table 5 in [71]) and permitted us to account for individual variability whereas the second one (trial LMM) provided a complementary trial-by-trial analysis and allowed us to consider both temporal and individual variability. Fixed effects variables were Group and Trial (numeric) with Participant as the random effects variable. We also added an autocorrelation structure of order 1, in the form of Trial nested within Participant. Model comparisons were done using the AIC and Chisq. Once again, a type III ANOVA table was obtained with Kenward-Roger “F” test statistics and Satterthwaite degrees of freedom, with Trial as the within-subject factor and Group as the between-subject factor (*F* and *p* values were reported, confidence level set to 0.95). Once again residual plots were visually inspected for deviations from homoscedasticity or normality in any measure. In this analysis, cortical excitability was approximated through a main effect of Group. In turn, habituation was confirmed in the presence of a main effect of Block (decrease confirmed through visual inspection).

##### Correlation analyses

The relationships between the continuous variables of age, migraine frequency (headache days/month; EM only), sensory sensitivity (SPQ Vision scores), cortical excitability (first block N1-P1 amplitude difference), and habituation (last block N1-P1 – first block N1-P1) were assessed using Spearman correlations. Correlation values (*r*) and* p* values were reported, and *p* values were adjusted for multiple comparisons using the FDR method.

## Results

### Participant demographics and migraine characteristics

Post-EEG recording, two participants were excluded due to technical problems (one HC, one EM), four due to severe depression (four EM), seven due to screening failure (seven EM), and five EM for being outside of the interictal phase. The final sample consisted of 18 EM (six patients reported aura as an accompanying symptom of migraine) and 27 HC. Groups were age- and gender-matched. No significant differences between patients with and without accompanying symptoms of aura were found, in terms of sensory sensitivity (SPQ Vision scale; *t*(15) = -1.037, *p* = 0.316), cortical excitability (first block N1-P1 amplitude difference; *t*(16) = 0.150, *p* = 0.883), and/or habituation (last block N1-P1 amplitude difference – first block N1-P1 amplitude difference; *t*(16) = -0.757, *p* = 0.460). For this reason, patients with and without accompanying symptoms of aura were collapsed for further analyses. Scores on the psychiatric measures related to anxiety, attention deficit disorder, and depression did not yield any significant differences between groups (see Table 3). Despite their relatively low headache frequency, patients reported mild to moderate disability and some impact of headache, according to the results of the clinical questionnaires (see Table 3).

**Table 3:** Statistical comparisons, between patients with episodic migraine (EM) and healthy controls (HC), of anthropometric, clinical, and psychiatric variables, for Experiments 1 and 2

	**Experiment 1**			**Experiment 2**		
Variable	**HC**	**EM**	***p***	**HC**	**EM**	***p***
N	27	18		29	19	
Gender (% of females)	100.0	100.0		82.8	84.2	1.00
Age (years old)	21.8 ± 2.03	22.8 ± 1.89	0.10	39.2 ± 8.84	40.9 ± 8.68	0.53
STAI-State (score)	12.2 ± 6.20	11.1 ± 5.90	0.53	5.0 [7.50]	11.0 [6.00]	0.002*
STAI-Trait (score)	20.4 ± 6.73	21.1 ± 7.96	0.76	12.0 [7.00]	19.0 [7.50]	0.001*
BDI-II (score)	2.0 [5.00]	3.0 [3.00]	0.90	2.0 [6.00]	6.0 [4.50]	0.003*
ASRS (score)	1.0 [3.00]	1.0 [2.00]	0.79	1.0 [2.00]	1.0 [2.00]	0.80
Migraine frequency (headache days/month)	NA	5.1 ± 3.24		NA	12.7 ± 4.03	
MIDAS (score)	NA	7.0 [8.75]		NA	52.3 ± 27.89	
HIT-6 (score)	NA	54.1 ± 5.99		NA	62.1 ± 5.67	
MSQ (score)	NA	30.4 [11.60]		NA	53.0 ± 14.13	
Accompanying symptoms of headache (% of participants)						
Aura	NA	31.6		NA	26.3	
Subjective presence of photophobia	NA	52.6		NA		
Subjective presence of phonophobia	NA	52.6		NA		
Nausea/vomiting	NA	31.6		NA		
SPQ Total (score)	116.1 ± 18.3	108.9 ± 17.0	0.20	110.3 ± 23.2	106.5 ± 23.3	0.58
SPQ Vision (score)	31.4 ± 5.08	27.4 ± 4.36	0.01*	30.5 ± 4.96	26.2 ± 4.05	0.003*
SPQ Motion (score)	7.7 ± 1.80	7.2 ± 1.99	0.46	7.0 [1.00]	7.0 [2.50]	0.36
SPQ Brightness (score)	6.0 ± 1.74	4.6 ± 1.77	0.02*	5.0 [2.00]	4.0 [3.00]	0.01*
SPQ Color (score)	6.0 [1.50]	5.0 [3.00]	0.03*	5.0 [2.00]	5.0 [1.00]	0.19
SPQ Acuity (score)	21.0 ± 2.92	10.0 [2.00]	0.09	12.0 [3.00]	10.0 [2.50]	0.01*

Means and standard deviations (mean ± SD) were used to report continuous normally distributed variables whereas medians and interquartile ranges (median [IQR]) were used for not normally distributed variables. To assess the null hypotheses, two-sided, unpaired t-tests of equal variance and two-sided, non-parametric Mann–Whitney U tests were used, respectively. Bold values with* were used to indicate significant differences between groups

### Sensory perception questionnaire

Results on the SPQ indicated that EM patients had increased hypersensitivity on the Vision subscale and in particular on Vision-Brightness and Vision-Color, as compared to HC (see Table 3). No other subscales related to Vision, as well as the Total SPQ score, yielded significant differences between groups. Please note that one participant did not complete the SPQ and therefore only data from 17 patients were included in this analysis.

### Electrophysiological analyses

#### Classic block analyses

The results of the ANOVA indicated a main effect of Block (*F*(1,43) = 8.895, *p* = 0.005) but no main effect of Group (*F*(1,43) = 2.279, *p* = 0.138) or significant Block x Group interaction (*F*(1,43) = 0.497, *p* = 0.485). The resulting main effect of Block would provide support to the presence of habituation in both groups (see Fig. 1B and C for a visual representation). Furthermore, given a lack of significant Group and interaction effects no significant differences in either cortical excitability or habituation were found between participant groups.

#### Block linear mixed-effects model

Here, we fitted a LMM to our data to account for individual variability. Extreme outlier trials were removed prior to fitting the model and were identified as any trial that was three times the interquartile range above the third and below the first quartile (11 trials total). The model, which best fit our data, following AIC and Chisq comparisons, was:

$$\begin{array}{c}N1\text{-}P1 \sim Block * Group,random = \sim 1 | Participant, \\ correlation = corAR1(form = \sim Trial | Participant)\end{array}$$

**Fig. 2:**
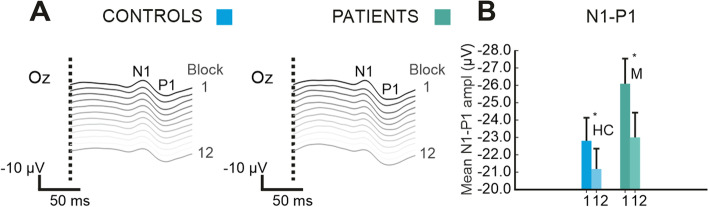
Visual illustration of both the block and linear mixed-effects models (LMMs) data from Experiments 1 and 2 with Block and Trial number on the x-axis and the N1-P1 peak-to-peak amplitude difference voltage on the y-axis. **A** Block LMM data, for both groups (green EM, blue HC) in Experiment 1. **B** Trial LMM data, for both groups (same colors) in Experiment 1. **C** Block LMM data, for both groups (same colors) in Experiment 2. **D** Trial LMM data, for both groups (same colors) in Experiment 2. Please note, that trials were grouped into bins of ten trials for both trial models (**B** and **D**) to facilitate visual inspection by reducing trial-to-trial variability

Results yielded a significant main effect of Block (*F*(1,7963) = 11.499, *p* = 0.0007) but no main effect of Group (*F*(1, 43) = 2.710, *p* = 0.100), or Block x Group interaction (*F*(1,7963) = 2.287, *p* = 0.130). The significant main effect of Block, in line with the classic analyses, continued to confirm the presence of habituation in both groups, through a significant decrease in N1-P1 amplitude over time (see Fig. 2A). Meanwhile, the lack of a main effect of Group or a significant Block x Group interaction indicated that EM and HC did not significantly differ regarding N1-P1 amplitude and by extension habituation and cortical excitability (confirmed through a visual inspection of Fig. 2A).

#### Trial linear mixed-effects model

Next, we used the Trial model to further increase our ability to account for both individual and temporal variability. To remove extreme trial outliers, we used the same criteria as for the Block analysis. The following model was determined to be optimal post-model comparisons:

$$\begin{array}{c}N1\text{-}P1 \sim Trial* Group,random = \sim 1|Participant, \\ correlation = corAR1(form = \sim Trial | Participant)\end{array}$$

In this case, the ANOVA yielded a significant main effect of Trial (*F*(1,24056) = 228.601, *p* = 2 × 10^–16^), no main effect of Group (*F*(1,43) = 1.954, *p* = 0.162), and no significant interaction (Trial x Group: *F*(1,24056) = 1.772, *p* = 0.183). This is consistent with the results obtained with the Block model and would support a lack of differences in habituation and cortical excitability between groups (confirmed through a visual inspection of Fig. 2B).

### Correlation analyses

Given our interest in the effect of age and headache frequency on the sensory processes under examination in this research study, we wanted to see whether sensory sensitivity scores, cortical excitability measures as defined by first block N1-P1 amplitude difference, or habituation (Block 6 N1-P1 amplitude – Block 1 N1-P1 amplitude) were correlated with age or disease severity (as quantified by the number of headache days/month) or amongst themselves. In the case of HC, age was not correlated with any of the three variables (see Table 4 for a full breakdown of *r* and FDR-corrected *p* values). Additionally, sensory sensitivity scores, cortical excitability, and habituation were not correlated with each other for the HC group. On the other hand, when correlations were run taking into account EM, a significant positive correlation between age and SPQ Vision was found. Furthermore, we also checked whether sensory sensitivity (SPQ Vision score), cortical excitability (first block N1-P1 peak-to-peak amplitude difference), and habituation (Block 6 N1-P1 peak-to-peak amplitude difference – Block 1 N1-P1 peak-to-peak amplitude difference) were correlated with each other and found a significant negative correlation between cortical excitability and habituation (see Table 4 for a full breakdown of *r* and FDR-corrected *p* values). In other words, patients with a lower first block amplitude (cortical hypoexcitability) had less of a difference between Block 6 and Block 1, which may indicate less habituation. On the other hand, patients with a greater first block amplitude (cortical hyperexcitability) had a greater difference between Block 6 and Block 1, indicating more habituation. None of the other variables were significantly correlated.

**Table 4:** Results of Spearman correlation tests to assess the association between age, headache frequency (migraine patients only), sensory sensitivity (SPQ Vision score), cortical excitability (first block N1-P1 peak-to-peak amplitude difference), and habituation (Block 6 N1-P1 peak-to-peak amplitude difference – Block 1 N1-P1 peak-to-peak amplitude difference) in Experiment 1

	**Experiment 1**					
**Group**	**Variable**	**Age**	**Headache Days**	**SPQ Vision**	**First Block Amplitude**	**Habituation**
**EM**	**Age**	-	0.52 (*.060*)	0.70 (.005*)	0.20 (*.501*)	-0.23 (*.501*)
	**Headache Days**	0.52 (*.060*)	-	0.30 (*.408*)	0.38 (*.232*)	-0.19 (*.501*)
	**SPQ**	0.70 (.005*)	0.30 (*.408*)	-	0.13 (*.632*)	0.19 (*.501*)
	**First Block Amplitude**	0.20 (*.501*)	0.38 (*.232*)	0.13 (*.632*)	-	-0.72 (.003*)
	**Habituation**	-0.23 (*.501*)	-0.19 (*.501*)	0.19 (*.501*)	-0.72 (.003*)	-
**HC**	**Age**	-	NA	0.14 (*.654*)	-0.20 (*.524*)	0.10 (*.718*)
	**SPQ Vision**	0.14 (*.654*)	NA	-	0.21 (*.524*)	0.06 (*.773*)
	**First Block Amplitude**	-0.20 (*.524*)	NA	0.21 (*.524*)	-	-0.39 (*.116*)
	**Habituation**	0.10 (*.718*)	NA	0.14 (*.654*)	-0.39 (*.116*)	-

The False Discovery Rate (FDR) was applied to adjust for multiple comparisons. The resulting *r* as well as the adjusted *p* values (*p*_adj_) are reported *r* (*p*_*adj*_ *)* with significant values indicated in bold*

## Experiment 2

### Method

#### Participants

Sixty-six participants with normal or corrected-to-normal vision, between 18 and 65 years old, were included and consisted of 36 middle-aged patients with EM (diagnosed by a neurologist using ICHD-3 criteria [5]) and 30 age- and gender-matched HC. Inclusion criteria were similar to Experiment 1 except for the recruitment location (specialized Headache Clinic) and disease severity (higher headache frequency). According to the results of Welch’s *t*-tests, EM in Experiment 2 were significantly older (*t*(19.786) = -8.867,* p* = 2.51 × 10^–8^) and had a significantly higher headache frequency (headache days/month: *t*(35) = -3.043,* p* = 0.004) than EM in Experiment 1. Given that the results from Experiment 1 did not confirm the previously described habituation deficit in patients with interictal, episodic migraine using either traditional analysis methods or newly implemented LMMs, we decided to run a second experiment with patients that were older and had a higher migraine frequency, to see whether this absence of significant effects continued to occur. Exclusion criteria were the same as in Experiment 1.

#### Procedure and paradigm

The procedure was very similar to Experiment 1, with participants answering the same questionnaires and completing an EEG recording comprised of a 5-min resting state and a subsequent PR task. Experimental stimuli were programmed and presented with custom-made scripts run on MATLAB R2017a (The Mathworks Inc., 2017) and Psychophysics Toolbox Version 3.0.13 [116, 117], running on Windows 10. All stimuli were presented on a BenQ XL2411P monitor with a screen size of 0.3 m height and 0.53 m width (CRT screen, resolution: 1024 × 768, 120 Hz refresh rate, background luminance: 21 cd/m2). Accurate timing was confirmed using the Black Box Toolkit (Black Box Toolkit, Ltd., Sheffield, UK).

Stimulus parameters were practically the same as in Experiment 1, with the exception of the reversal rate (3.1 Hz) and the number of blocks (12 blocks of 100 trials, divided post-recording). The reversal rate was incremented given that some authors have proposed that increasing this measure can help to detect the lack of habituation reported in migraine [71, 123]. Furthermore, several studies reporting a deficit of habituation used a reversal rate of 3.1 Hz [17, 19, 20, 52].

#### EEG recording

Using a BrainAmp32 Standard amplifier and a BrainVision recorder polybox BP-BM-30 actiCAP32, continuous EEG recordings (digitized, 1000 Hz sampling rate, 50 Hz online notch filter) were collected (Brain Products GmbH). The 32 active electrodes were placed in standard positions on an elastic cap. The online reference electrode was placed on the tip of the nose, and the ground electrode was inserted at the AFz point in the cap. Left and right mastoids as well as vertical and horizontal electrooculograms, were used as external electrodes. Impedances were kept below 15 kΩ.

#### EEG pre-processing

The same procedure was used as in Experiment 1, with the exception being that: no notch filter was applied offline as a notch filter was used during acquisition and epoch segments were from 0 to 150 ms. The metrics for the FIR band-pass filters (zero-phase) in Experiment 2 for the high-pass and low-pass filters are detailed upon continuation (high-pass: Frequency 0.1 Hz, order 33001, cutoff -6 dB; low-pass: frequency 60 Hz, order 221, cutoff -6 dB).

#### Analyses

##### Psychiatric, clinical, and SPQ questionnaires

Analyses were the same as in Experiment 1.

##### Electrophysiological data

*PR-VEP.*  For all electrophysiological analyses, the same procedure was used as in Experiment 1, with a focus on the N1-P1 peak-to-peak amplitude difference at the Oz electrode. The only difference was that we considered 12 blocks and 1200 trials. Each block contained a maximum of 100 trials with a mean of 93.72 ± 6.912 (range: 56–100) clean trials per participant. Participants had a grand mean of 1124.58 ± 57.203 trials included post artifact rejection, out of a total of 1200 (range: 971–1197). Components were identified in the same way as in Experiment 1. In this case, N1 was the most negative peak between 73–101 ms (peak at 88 ms and window of ± 15 ms), and P1 was the most positive peak between 96–136 ms (peak at 116 ms and window of ± 20 ms).

##### Classic block analyses

Analyses were the same as in Experiment 1 with the exception that the factor Block consisted of Blocks 1 and 12. An additional analysis comparing Blocks 1 and 6 was also provided to ensure that the number of blocks did not have an effect on the results.

##### Block linear mixed-effects model

Analyses were the same as in Experiment 1 with the exception that the fixed-effects variable in the LMM termed Block consisted of Blocks 1 and 12 and the within-subject factor in the subsequent ANOVA also considered Blocks 1 and 12. The results assessing only Blocks 1 and 6 were also reported.

##### Trial linear mixed-effects model

Analyses were the same as in Experiment 1 with the exception that the fixed-effects variable Trial in the LMM consisted of Trials 1 to 1200, along with the within-subject factor Trial in the ANOVA (Trials 1 to 1200). Finally, an additional analysis examining Trials 1 to 600, to make sure that the number of trials did not have a significant influence on the results, was also reported.

##### Correlation analyses

Correlation analyses were carried out following the same methodology as in Experiment 1.

## Results

### Participant demographics and migraine characteristics

Five participants were excluded for the following reasons: three for technical problems (two EM, one HC) and two due to an insufficient number of clean trials following artifact rejection (two EM). We also had to exclude 17 EM for not being in the interictal phase, following the criteria discussed in the Method section. This resulted in a final sample of 19 EM patients (five had migraine with aura) and 29 HC. EM and HC were age- and gender-matched. Once again, patients with and without aura as an accompanying symptom did not significantly differ with regard to sensory sensitivity (*t*(14) = 1.412, *p* = 0.180), cortical excitability (*t*(14) = 0.232, *p* = 0.820), and/or habituation (*t*(14) = -1.965, *p* = 0.070). Therefore, patients were collapsed for further analyses. As expected in a sample of patients with heightened disease severity, scores related to anxiety and depression were significantly elevated in EM as compared to HC. In contrast, attention deficit disorder scores did not vary between groups. Furthermore, patients reported severe disability and severe headache impact as measured by the clinical questionnaires. See Table 3 for statistical comparisons of demographic and migraine characteristic data.

### Sensory perception questionnaire

Similarly, to Experiment 1, patients with EM reported significant hypersensitivity on the Sensory Perception Questionnaire as compared to HC, on Vision, Vision-Brightness, and Vision-Acuity but not on the total score, Vision-Motion, or Vision-Color (see Table 3).

### Electrophysiological analyses

#### Classic block analysis

**Fig. 3:**
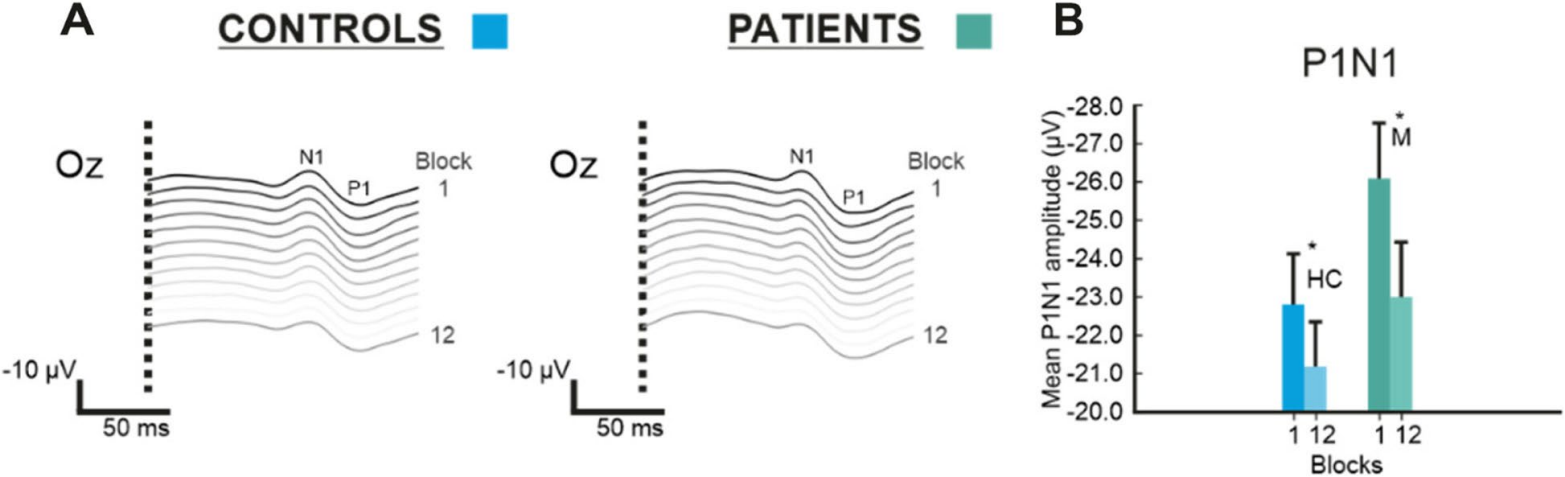
Resulting visual evoked potentials (VEPs) and habituation to pattern-reversal stimulation in Experiment 2. **A** VEPs at the Oz electrode, with time (in ms) on the x-axis and N1-P1 peak-to-peak amplitude difference voltage on the y-axis, for each block (1 to 12) during the Pattern-Reversal task, and both groups (EM right, HC left). **B** Bar graph with Block number on the x-axis and mean N1-P1 peak-to-peak amplitude difference voltage on the y-axis. Habituation of the N1-P1 between Blocks 1 and 12 (green EM, blue HC)

The type III two-way mixed ANOVA results on N1-P1 amplitude data were examined and yielded a main effect of Block (*F*(1,46) = 24.082, *p* = 1.2 × 10^–5^), no main effect of Group (*F*(1,46) = 0.872, *p* = 0.355), and no significant Block x Group interaction (*F*(1,46) = 2.384, *p* = 0.129). These results would suggest habituation in both groups as supported by the main effect of Block (see Fig. 3). Furthermore, a lack of significant differences between groups with regard to cortical excitability were also found, supported by an absence of significant Group or Block x Group interaction (see Fig. 3).

Furthermore, to verify that the number of blocks did not influence the results, we also assessed what happened at Block 6 (similarly to Experiment 1). Similarly to the analysis using Blocks 1 and 12, we found a main effect of Block (*F*(1,46) = 6.603, *p* = 0.013) but no main effect of Group (*F*(1,46) = 1.012, *p* = 0.320) or significant Block x Group interaction (*F*(1,46) = 1.255, *p* = 0.268). Therefore, the number of Blocks did not appear to significantly affect the results.

#### Block linear mixed-effects model

Extreme outlier trials were removed prior to fitting the model and were identified as any trial that was ± three times the interquartile range (35 trials total). Only data from Blocks 1 and 12 were used. After performing model comparisons, the final model that best fit our data was:

$$\begin{array}{c}N1\text{-}P1 \sim Block * Group,random = \sim 1 | Participant,\\ correlation = corAR1(form = \sim Trial | Participant)\end{array}$$

First, a significant main effect of Block (*F*(1,8888) = 39.680, *p* = 2.992 × 10^–10^) but no significant main effect of Group (*F*(1,46) = 1.478, *p* = 0.224) was found. The interaction between Block x Group was also significant (*F*(1,8888) = 14.371,* p* = 1.501 × 10^–4^) and was decomposed to further explore the results. When the N1-P1 amplitude was compared between Blocks 1 and 12 as a function of Group, both HC (*t* = 6.299, *p* < 0.0001) and EM (*t* = 9.941, *p* < 0.0001) showed a significant decrease in N1-P1 amplitude between Block 1 and Block 12. This would once again appear to indicate habituation in both groups (see Fig. 3B and C and Fig. 2C for a visual representation). Furthermore, N1-P1 amplitude at Block 1 and 12 was separately compared as a function of Group, with no significant differences between Groups found at either Block 1 (*t* = -1.216, p = 0.345) or at Block 12 (*t* = -0.659,* p* = 0.616). Please note that the lack of differences in Block 1, would appear to indicate a lack of significant differences in cortical excitability between EM and HC.

We also ran an additional analysis using only Blocks 1 and 6 (similarly to Experiment 1) to ascertain that the number of Blocks did not significantly affect the results. Once again, we found a main effect of Block (*F*(1,8888) = 5.6377, *p* = 0.018) but no main effect of Group (*F*(1,46) = 1.436, *p* = 0.231). This time, the Block x Group interaction proved significant (*F*(1,8888) = 8.140, *p* = 0.004). When we decomposed this interaction, we found a significant difference between Blocks 1 and Block 6 for both EM (*t* = 5.559, *p* < 0.0001) and a trend for HC (*t* = 2.374, *p* = 0.053). The comparisons between EM and HC for Block 1 (*t* = -1.198. *p* = 0.355) and Block 6 (*t* = -0.804, *p* = 0.510) amplitude were not significant. The results mirror those reported at Block 12, mainly a lack of significant differences in cortical excitability and habituation between groups.

#### Trial linear mixed-effects model

The final model was the same as in Experiment 1.

$$\begin{array}{c}N1\text{-}P1 \sim Trial* Group,random = \sim 1|Participant, \\ correlation = corAR1(form = \sim Trial | Participant)\end{array}$$

The results from the two-way mixed ANOVA yielded a main effect of Trial (*F*(1,53803) = 264.649,* p* =  < 2 × 10^–16^), as well as a main effect of Group (*F*(1,46) = 5.529,* p* = 0.019), and a significant interaction between Trial x Group (*F*(1,53803) = 627.299,* p* =  < 2 × 10^–16^). The presence of a main effect of Trial would suggest differences between some trials, however this is not unexpected and habituation is likely, as supported by comparing the first trial amplitudes to the last ones in Fig. 2D. The significant main effect of Group and Trial x Group interaction, on the other hand, might suggest that patients exhibit some general hyperexcitability as compared to healthy controls as well as potential differences in the habituation slope. Furthermore, differences in the habituation slope are not indicative of a lack of habituation in EM, on the contrary, they show that the habituation slope is different between groups most likely due to the higher amplitude on certain trials in patients as compared to controls at the beginning of the task (see Fig. 2D). These results contrast with the findings from the block LMM.

Finally, we assessed the Trial model using Trials 1 to 600 and found no main effect of Trial (*F*(1,27003) = 0.164, *p* = 0.685) or Group (*F*(1,46) = 1.328, *p* = 0.249) but a significant Trial x Group interaction (*F*(1,27003) = 9.236, *p* = 0.002). The results of this analysis in particular, might indicate the need for more trials.

### Correlation analyses

With respect to the EM group, no significant correlations were found between age and/or migraine frequency and any of the measures of interest (i.e., sensory sensitivity (SPQ Vision score), cortical excitability (first block N1-P1 peak-to-peak amplitude difference), and/or habituation (Block 6 N1-P1 peak-to-peak amplitude difference – Block 1 N1-P1 peak-to-peak amplitude difference), see Table 5 for *r* and FDR-corrected *p* values). The three variables (omitting age and/or migraine frequency) were also not correlated amongst each other (see Table 5 for *r* and FDR-corrected *p* values). On the other hand, with regard to HC, age was not correlated with either sensory sensitivity, cortical excitability, and/or habituation (see Table 5 for *r* and FDR-corrected *p* values). However, cortical excitability and habituation were significantly negatively correlated, similarly to what we saw with patients in Experiment 1 (see Table 5 for *r* and FDR-corrected *p* values).

**Table 5:** Results of Spearman correlation tests to assess the association between age, headache frequency (migraine patients only), sensory sensitivity (SPQ Vision score), cortical excitability (first block N1-P1 peak-to-peak amplitude difference), and habituation (Block 12 N1-P1 peak-to-peak amplitude difference – Block 1 N1-P1 peak-to-peak amplitude difference) in Experiment 2

**Experiment 2**						
**Group**	**Variable**	**Age**	**Headache Days**	**SPQ Vision**	**First Block Amplitude**	**Habituation**
**EM**	**Age**	-	0.05 (*.994*)	0.02 (*.994*)	0.00 (*.994*)	0.22 (*.684*)
	**Headache Days**	0.05 (*.994*)	-	-0.07 (*.994*)	-0.27 (*.684*)	0.05 (*.994*)
	**SPQ Vision**	0.02 (*.994*)	-0.07 (*.994*)	-	0.20 (*.684*)	0.25 (*.684*)
	**First Block Amplitude**	0.00 (*.994*)	-0.27 (*.684*)	0.20 (*.684*)	-	-0.25 (.684)
	**Habituation**	0.22 (*.684*)	0.05 (*.994*)	0.25 (*.684*)	-0.25 (.684)	-
**HC**	**Age**	-	NA	-0.16 (*.541*)	-0.34 (*.135*)	0.13 (*.541*)
	**SPQ Vision**	-0.16 (*.541*)	NA	-	0.12 (*.541*)	-0.28 (*.237*)
	**First Block Amplitude**	-0.34 (*.135*)	NA	0.12 (*.541*)	-	-0.55 (.005*)
	**Habituation**	0.13 (*.541*)	NA	-0.28 (*.237*)	-0.55 (.005*)	-

The False Discovery Rate (FDR) was applied to adjust for multiple comparisons. The resulting *r* and adjusted *p* values (*p*_adj_) are reported *r* (*p*_*adj*_*)* with significant values indicated in bold*

## Discussion

The objective of our study was to explore visual sensitivity (using the SPQ) as well as cortical excitability and habituation (both measured with PR-VEPs), as a function of age and disease severity. Two samples of patients with episodic migraine and their headache-free controls were used. The first consisted of a group of young adults with EM and the second a middle-aged group of EM patients. The results of both experiments yielded three main findings: (i) significant hypersensitivity, as seen by lower scores on the SPQ Vision scale in EM as compared to HC, (ii) no significant differences in cortical excitability or specifically N1-P1 first block peak-to-peak amplitudes in EM and HC and, (iii) no deficit of habituation, evidenced by habituation of the N1-P1 amplitude across blocks in both EM and HC.

### Visual sensitivity

Hypersensitivity to visual stimuli has been found to occur in patients with migraine, both ictally and interictally, and has been measured using different methods including self-report questionnaires and sensory thresholds [9, 12–14]. In this study, we selected the SPQ self-report questionnaire, a validated instrument for exploring self-reported sensory sensitivity, given its use in neurological and pain research [16, 124, 125]. In our study, we found significantly lower values on the Vision scale of the SPQ in both Experiment 1 and Experiment 2 in EM as compared to HC, denoting a general hypersensitivity to visual stimuli in patients. These results are consistent with what is found in the clinical setting, where patients frequently complain of alterations in sensory processing, including ictal and interictal sensitivities to light, as well as discomfort to certain patterns, colors, and contrasts [126, 127]. They are also in line with studies using self-report measures, indicating that migraine patients regularly report a greater number of visual sensitivities in their environment when compared to non-headache controls [6], as well as increased light sensitivity when exposed to the same stimuli of varying intensity [13]. Furthermore, and perhaps most convincingly, psychophysical studies of sensory discomfort thresholds in migraine have yielded both ictal and interictal differences in patients as compared to healthy controls, with patients demonstrating a hypersensitivity to visual stimuli, as seen through decreased visual discomfort thresholds [9, 12, 14]. The results of this study also yielded a significant positive correlation between age and sensory sensitivity (SPQ vision score), which would be in line with results from research on photophobia and phonophobia in migraine, indicating an increase of these sensory alterations with age [98]. We did not find these results in Experiment 2, with the older sample of patients, which perhaps may be indicative that age-related changes in sensory sensitivity tend to flatten out with age, which is consistent with studies indicating that in older patients (60 +) there is even a reported decrease in photophobia and phonophobia [99], most likely related to the degeneration of sensory receptors [104] (for a review see [105]). Another possible explanation may be that the difference is more headache-disability based and that in patients with more headaches other predictors matter more than age. Moreover, given that we found the same effect in both experiments and also taking into account previous literature [6, 9, 12–14, 126], we would propose that the presence of interictal visual hypersensitivity in migraine patients appears to be quite robust.

### Cortical excitability

The PR task, coupled with EEG, has been suggested as a good tool to measure a variety of sensory cortical properties, including excitability and habituation. Regarding cortical excitability in migraine, during the interictal period, there exist two predominant theories. First, a theory of hypoexcitability, or a reduced preactivation level of sensory cortices [18, 128], has garnished growing support in recent years. In fact, reduced preactivation levels might be linked to thalamocortical dysrhythmia in patients, which may ultimately result in a lack of habituation or even potentiation [18]. Second, a theory of hyperexcitability [129, 130], postulates the opposite and is thought to be a consequence of either increased neuronal excitation or decreased inhibition (see [131] for a review). Despite numerous studies using a variety of paradigms, the results remain controversial with certain studies pointing to reduced inhibition [128] and others to heightened excitation [132]. Given that results appear to be quite divided, some authors have proposed the broader term of “cortical dysexcitability” to encompass possible alterations of cortical excitability in patients with migraine [133].

In the present research study, no significant differences in the N1-P1 peak-to-peak amplitude between EM and HC were found in either experiment at any of the first blocks. Our finding adds to a body of evidence in the literature on PR tasks in patients with migraine, which has encountered non-significant differences between this clinical population interictally and healthy controls [19, 20, 35] (for a review see [133]). Currently, and considering the results of our study, it remains difficult to establish a clear picture regarding cortical excitability in migraine patients. The most plausible explanation is that the competing theories coexist, hinting at different profiles of cortical excitability that may affect patients’ electrophysiological responses. In fact, the sum of both profiles may lead to the lack of significant differences, such as the ones found here, in certain samples when compared to healthy controls. In sum, it is not possible to completely discard the hypothesis that migraine patients could have normal cortical excitability, during the interictal period.

### Habituation

Despite certain controversy [21, 31, 32, 35, 71, 77], an interictal deficit of habituation has been proposed as a hallmark of migraine electrophysiology [134, 135], supported by some past studies [18–20, 52, 70]. However, in both experimental EM groups, we did not find the anticipated deficit of habituation interictally in the amplitude of the N1-P1 peak-to-peak. In particular, patients continued to habituate and, in Experiment 2, even showed steeper habituation slopes as compared to controls, indicating more pronounced habituation. This result is not completely unexpected and adds to a growing body of literature reporting no habituation deficit in patients [21, 31, 32, 35], as well as a lack of replicability of the interictal habituation deficit, reported in patients with migraine [77, 136–138]. These results do not discard that a habituation deficit might be present in specific migraine patients [30] under specific conditions [69, 123], but given the presence of negative results in several studies, perhaps it should not be considered as a defining and general characteristic of migraine, at least in the visual domain. One potential explanation for a lack of significant results could be related to the characteristics of the sample (at least in Experiment 1), in that young patients have more metabolic resources [139, 140], which compensate for visual effort [141], making it more difficult to induce a habituation deficit. Nonetheless, even with a sample of middle-aged patients with increased disease severity, we did not find the anticipated deficit of habituation despite presumably decreased metabolic resources in this sample. Another possibility is that the habituation deficit exists but the stimulation being used, in this case, the PR, does not adequately reproduce real-world conditions.

We also found that in the EM group in Experiment 1, decreased cortical excitability (lower preactivation levels) was correlated with less habituation (and perhaps even potentiation). This is in line with several past studies [17, 18, 30, 57] that also showed that first block amplitude was negatively correlated with habituation. In fact, in a study by Coppola et al. [70], the authors proposed that these lower preactivation levels may be indicative of a hypothesized thalamocortical dysrhythmia. Interestingly, the findings could also be compatible with the ceiling theory based on Knott and Irwin [142] and applied to migraine [143], which postulates that interictally diminished pre-activation excitability levels of the sensory cortices may be related to the reported deficit of habituation in migraine. However, these explanations remain speculative given that this correlation between cortical excitability and habituation was not present in EM in Experiment 2.

### Age and migraine frequency

Keeping in mind the above-mentioned concepts, we wanted to see whether a relationship between hypersensitivity and hyperexcitability and/or habituation might be modulated by factors related to age and migraine frequency. In recent years, it has been well-documented that the relationship between age and migraine frequency tends to follow an inverse U-shaped curve, in that migraine frequency usually increases with age, reaching a peak and then declining with older age, although this is not always the case. In episodic migraine, peak prevalence tends to occur between 30–39 years old [96]. Importantly, the sensory sensitivity profile follows a similar curve, in that, patients with migraine as compared to healthy controls, tend to report more hypersensitivity with increased age and migraine frequency, indicated by an increase in the mean number of visual stressors peaking around 46–60 years old [6], and then progressively declining as of 50 years old [100]. Other studies, looking at the way in which visual sensitivity changes with migraine frequency/age, found that interictal photophobia also appears to be correlated to migraine frequency, according to self-perception reports (age range: 18–55 years old; [144]) and photophobia scores (age range: 20–79 years old; [145], despite [146]). These results would support a positive association between visual sensitivity and migraine frequency, with increased disease severity being linked to heightened sensitivity.

Recently, it has been proposed that high-frequency episodic migraine patients may in fact be more clinically similar to chronic migraine patients than low-frequency episodic migraine patients and symptomatology, such as visual sensitivity, may be modulated similarly [147]. In our study, significant differences in visual hypersensitivity between EM and HC were found in both experiments, although this variable was only found to be related to age in Experiment 1 and was not correlated to migraine frequency in either experiment, according to the results of the correlation analyses. Most likely, the absence of a significant correlation between these measures was influenced by the small sample sizes (see Limitations section) but also by the homogeneity amongst the participants in each group.

Pertaining to cortical excitability and habituation in migraine patients as compared to healthy controls, to the best of our knowledge, no study has directly evaluated, using PR-VEPs their relationship to age and migraine frequency. In this light, a recent meta-analysis has highlighted an important lack of information in several papers (see [120] for list and meta-analysis criteria), which made it impossible to effectively evaluate the effect of migraine frequency on the amplitude and habituation of VEPs. Age was also discussed as a limiting factor by the same authors, to the generalization of results and they proposed that future studies should take heed to consider the effects of age on VEP attenuation [120]. Considering the lack of significant differences between patients with migraine and healthy controls and correlations between these variables in our research study, the relationship between migraine frequency, age, and cortical measures such as excitability and habituation, remains elusive.

### Methodological considerations

Pertaining to PR-VEPs, several authors have highlighted the difficulty in establishing clear findings when each study uses vastly different methodological parameters, clinical samples, interictal criteria, and statistical analyses, as well as differences in blinding and task instructions. Additionally, within the literature itself, the terminology used to describe stimulation parameters is inconsistent. For example, with regard to temporal frequency, some studies use reversals/second [18, 61, 134] whereas others use Hz [17, 34, 52] and these terms do not necessarily mean the same thing across studies making it difficult to assess results and interpret, which parameters are more or less commonly used. To avoid these problems in the future, we would recommend researchers to select one metric, for example Hz, to be used accurately and consistently across studies. In our study, we selected stimulus parameters based on recommendations from previous authors [21, 32, 69, 71, 77, 123]. With regard to statistical analyses, we used a LMM approach that was methodologically superior to previously used analyses (such as repeated measures ANOVAs), to see whether this would provide more precision in uncovering subtle group differences, while also running the classic analysis methods as control analyses. Our study is, to the best of our knowledge, one of the few to analyze PR data in migraine using a statistical model approach [86, 148]. Past research studies have primarily made use of least squares slopes, linear regression slopes, or repeated measures ANOVAs of amplitude, among other methods, to study cortical excitability and habituation [30]. Compared to these methods, LMMs hold several advantages, particularly in studies of a clinical nature. First, and most importantly, all of the information and variability in the data are preserved in LMMs, especially with regard to individual and temporal factors [149, 150]. This is particularly important, given that EEG applications introduce a higher degree of complexity to the data. Furthermore, LMMs offer a superior approach to handling differences in the number of individual values (missing data), dropout in longitudinal studies, and are more robust when dealing with a smaller number of observations and/or unbalanced data [149, 150]. Given that EEG studies often carry high variability due to the nature of electrophysiological artifacts and their impact on the number of trials included in the final analysis, LMMs offer a statistically-sound approach to deal with these discrepancies [151]. Also, migraine patients tend to be quite heterogeneous [152], therefore the use of LMMs also helps to account for within-participant differences, which are often unaccounted for in traditional analyses. However, even with a statistically more powerful method, we still found negative results for both cortical excitability and habituation apart from the increased cortical excitability for the trial LMM in Experiment 2.

### Relationship between sensory sensitivity, cortical excitability, and habituation

Taking into account our results, it is interesting to reflect on the apparent dissociation of the three processes of sensory sensitivity, cortical excitability, and habituation. The question remains whether hypersensitive individuals also showcase differences at a neural level, in terms of brain responses. Research on habituation and sensory sensitivity in other clinical disorders, such as obsessive–compulsive disorder and autism, would appear to suggest that deficits in habituation may reduce an individual’s ability to suppress stimuli, which may lead to the development of hypersensitivities [153, 154]. However, in the literature on migraine, only a few studies have examined the relationship between these processes and did not find an association between visual evoked potentials and sensory measures such as visual discomfort thresholds [35]. This would support the notion that a direct link between EEG and behavior is often missing and difficult to rationalize. That being said, it remains of interest to continue investigating whether the subjective experiences reported by patients with migraine as they relate to sensory perception and their subsequent impact on behavior can be connected to a more objective neural measure, especially given that these processes appear to share a link. Perhaps, cortical excitability and habituation measures cannot explain the sensory sensitivity commonly reported by patients or maybe PR-VEPs are simply unable to tap into these processes with sufficient adequacy so as to provide a tool to study a potential relationship between them.

### Limitations

The main limitation was the fact that our visual stimulus used for the EEG recording was unable to measure sensory sensitivity directly, unlike our variables of cortical excitability and habituation. However, despite this limitation the PR task was chosen given its widespread use in studying visual processing in migraine patients and the ability to carefully select stimulation parameters based on recommendations from previous studies [21, 32, 69, 71, 77, 123]. In the future, it would be important to search for a paradigm that would permit us to evaluate all three concepts simultaneously. Also, stimulating with two different reversal rates may limit the ability to compare the results of both experiments amongst each other as well as to the literature. The results using the 3.1 Hz temporal frequency can be compared to some studies [17, 19, 20] whereas those using 1.55 Hz can be assessed with respect to others [61, 70, 72], although not at the same time. Nevertheless, although the results of both Experiments may not be directly comparable, they can be evaluated with respect to previous literature given that both reversal rates have been used in the past and have been found to yield both positive and negative results regarding a deficit of habituation in patients with episodic migraine interictally. In fact, the results of our study are in line with those of others [21, 32, 71, 77] that used different reversal rates and were also unable to reproduce the anticipated deficit of habituation. This would be consistent with the hypothesis proposed by Omland et al. [77] that different stimulation parameters are unable to explain the discrepant findings in previous VEP studies, which may help us clarify where the differences in the literature arise from. Likewise, we chose to use binocular stimulation, which may limit our ability to compare our results to past literature, although it is important to note that there are currently no set guidelines as far as habituation research in terms of monocular/binocular stimulation [155]. Additionally, although binocular stimulation could cause summation or subtraction phenomena in the signal affecting N1 and P1 latencies and amplitudes, Tobimatsu and Kato [156] found these effects to be more pronounced for the P50-N75 amplitude than the N1-P1 amplitude, which was the focus of the current study. Binocular summation is also not significant under transient conditions (1.5 Hz- 3.0 Hz), meaning that it should not be an issue in either Experiment 1 or 2 of this research study [157]. Finally, binocular stimulation has been previously used in with the Pattern-Reversal paradigm in patients with migraine [24, 31, 92, 158]. Furthermore, with regard to the correlation analyses, it is possible that the results were not significant due to small sample sizes and homogeneity in our participant groups. Also, another important aspect to take into account concerning age, migraine frequency, and cortical measures, is their relationship to gender. Migraine is about three times more frequent in women than in men and attacks tend to be more severe [159]. However, in the present study, we were unable to evaluate the interaction between gender, the previously mentioned factors, and our variables of interest. In Experiment 1, the entire sample consisted of women and in Experiment 2, the percentage of women and men was equivalent to that reported in the general population (approx. 3 to 1). Given that our groups were gender-matched, this avoided any potential distortions of our results. Furthermore, the effects of gender have not been accounted for in previous studies with regard to these measures, despite some studies indicating the presence of structural and functional brain differences in men and women, related to migraine [160]. For this reason, we highly encourage future studies to take into account gender and its effect on these variables. Finally, despite collecting information about aura, we did not expressly evaluate these concepts separating migraine patients into patients with and without aura due to the resulting small sample size. That being said, past literature reporting negative results with regard to cortical excitability and habituation were not limited to patients with migraine without aura, but also found normal cortical excitability and habituation in patients with migraine with aura [35, 71].

## Conclusions

In conclusion, both experiments indicated a significant hypersensitivity to visual stimuli in patients with EM interictally but no differences in either cortical excitability or habituation between groups. These results would provide support for two different things. With respect to these metrics, the alterations in patients may be less pronounced than originally anticipated. At the same time, our results also clearly highlight a necessity for the standardization of methodological parameters. Further research is required to precisely define the optimal parameters for assessing sensory sensitivity, cortical excitability, and habituation in different age groups and migraine subtypes, as well as, as a function of disease severity, and other factors. Doing so would be essential in resolving the debate as to the use of these metrics as potential biomarkers of migraine.

## Data Availability

The datasets used and/or analysed during the current study are available from the corresponding author on reasonable request.

## Abbreviations

ANOVA: Analysis of Variance
ASRS: ADHD Self-Report Scale
BDI-II: Beck Depression Inventory-II
EEG: Electroencephalography
EM: Episodic Migraine (Patients)
HC: Healthy Controls
HIT-6: Headache Impact Test-6
ICHD-3: International Classification of Headache Disorders 3rd edition
IHS: International Headache Society
LMM: Linear Mixed-effects Models
MIDAS: Migraine Disability Assessment Test
MSQ: Migraine-Specific Quality of Life Questionnaire
N1: N100 electrophysiological component
N1-P1: Peak-to-peak amplitude difference of N1-P1
P1: P100 electrophysiological component
PR: Pattern-Reversal
PR-VEP: Pattern-Reversal Visual Evoked Potential
SPQ: Sensory Perception Quotient
STAI: State-Trait Anxiety Inventory
VEP: Visual Evoked Potential
